# Microclimatic Alteration after Logging Affects the Growth of the Endangered Lichen *Lobaria pulmonaria*

**DOI:** 10.3390/plants11030295

**Published:** 2022-01-22

**Authors:** Luca Di Nuzzo, Paolo Giordani, Renato Benesperi, Giorgio Brunialti, Zuzana Fačkovcová, Luisa Frati, Juri Nascimbene, Sonia Ravera, Chiara Vallese, Luca Paoli, Elisabetta Bianchi

**Affiliations:** 1 Department of Biology, University of Florence, 50121 Florence, Italy; luca.dinuzzo@unifi.it (L.D.N.); renato.benesperi@unifi.it (R.B.); e.bianchi@unifi.it (E.B.); 2 DIFAR, University of Genoa, 16126 Genoa, Italy; giordani@difar.unige.it; 3 TerraData Environmetrics, Spin-Off Company of the University of Siena, 58025 Monterotondo Marittimo, Italy; brunialti@terradata.it (G.B.); frati@terradata.it (L.F.); 4 Plant Science and Biodiversity Centre, Slovak Academy of Sciences, 84523 Bratislava, Slovakia; zuzana.fackovcova@savba.sk; 5 Department of Biological, Geological and Environmental Sciences, University of Bologna, 40126 Bologna, Italy; juri.nascimbene@unibo.it (J.N.); vallese.chiara@gmail.com (C.V.); 6 Department of Biological, Chemical and Pharmaceutical Sciences and Technologies, University of Palermo, 90123 Palermo, Italy; sonia.ravera@unipa.it; 7 Department of Biology, University of Pisa, 56126 Pisa, Italy; 8 CIRSEC, Centro Interdipartimentale di Ricerca per lo Studio degli Effetti del Cambiamento Climatico dell’Università di Pisa, 56124 Pisa, Italy

**Keywords:** conservation, epiphytic lichens, forest management, growth rates, microclimate, translocation

## Abstract

Microclimatic conditions are important in determining lichen distribution at small scale, and may determine whether the species persist when the surrounding environmental conditions have drastically changed. This is the case with forest management, since a sudden variation of microclimatic conditions (increase of solar radiation, temperature, wind and a reduction of humidity) may occur after logging. In this study, the combined effect of forest logging and microclimatic conditions on the growth probabilities and growth rates of the model species *Lobaria pulmonaria* was assessed in mixed oak stands. To this purpose, 800 fragments of *L. pulmonaria* (<1 cm) were transplanted in logged and unlogged stands for two years. Young and adult fragments were positioned on Turkey oak boles according to distance from the ground (100 and 50 cm) and aspect (north and south). The results, evaluated by generalized linear mixed models on a yearly basis, highlighted differences in growth—particularly on isolated trees in the logged stand. South-exposed samples in the logged stand showed a low probability of growth, while samples transplanted north in the unlogged stand showed higher growth probabilities. However, the highest annual growth coefficients corresponded to south-exposed samples 50 cm from the ground in the unlogged stand. In general, higher growth rates were observed in young thallus fragments when compared with adult ones. Beyond confirming the importance of microclimate for lichen ecology, these results could be implemented in conservation actions to preserve *L. pulmonaria* populations in logged forests.

## 1. Introduction

Microclimatic conditions are important in determining lichen distribution at small scale and can affect the persistence of a species when the surrounding environmental conditions have drastically changed [1]. The structural complexity of forests creates heterogeneous microclimates at a fine scale, and the link between microclimatic features and their physiological and ecological importance, as well as their consequences for biodiversity conservation, have long been recognized [2]. Nevertheless, despite a deepening interest in how microclimatic parameters can reduce threats to understory species, there is still a gap in the consideration of microclimatic parameters when implementing conservation policies during forest management, in particular their use in maintaining suitable microclimatic refugia for target organisms [2,3]. 

Several studies have highlighted the negative impact of intensive forest logging on lichen communities, demonstrating a reduction of biodiversity and capacity to provide ecosystem services [4,5,6,7]. Gradients in microclimatic parameters, determined by forest logging along forest edges and according to the aspect of isolated trees, can affect several ecosystem functions, including evapotranspiration, nutrient and water availability and cycling, N and CO_2_ diffusion and photosynthetic processes [7,8,9,10], with implications on the survival and growth of various target organisms. In this regard, the important role of lichens in providing ecosystem services (habitat, shelter, food) and functions (nutrient and water cycling, metal chelation, microclimate regulation, primary colonization and soil formation) has been widely acknowledged [11].

As an example of an endangered forest lichen, *Lobaria pulmonaria* has been widely used as a model organism for population ecology and conservation biology. It is a tripartite foliose epiphytic species with a thallus often exceeding 20–30 cm in diameter, and has green algae as its main photobiont and nitrogen-fixing cyanobacteria within its cephalodia. It is considered a flag species for lichen conservation and also an umbrella species—a suitable sensitive indicator of forest habitats worthy of conservation and hosting other rare lichens (e.g., cyanolichens) [5,7,12,13]. The species has declined throughout Europe as a consequence of air pollution (especially past air pollution) and is threatened by intensive forest management, the effects of which are expected to be further exacerbated by climate change [14].

On the whole, silvicultural practices may especially threaten *L. pulmonaria* (and sensitive forest lichens in general) by causing habitat fragmentation, degradation and loss, with negative consequences on local population size, structure and dynamics [7,12,15]. As a primary consequence of logging (beyond the loss of the substrate), forest lichens are exposed to a sudden microclimatic variation, consisting of an increase in solar radiation, temperature and wind as well as a reduction of humidity. Such drier conditions, if in excess of the ecological range of the species, may negatively affect their photosynthetic activity, and hence their overall vitality [16,17]. These aspects can be exacerbated in potentially arid environments such as the Mediterranean region, where oak-dominated forests are one of the main habitats of *L. pulmonaria* populations [18]. The climatic niche of *L. pulmonaria* largely overlaps (>70%) with that of oak-dominated forests [19]; hence, it is important to maintain suitable refugia for the conservation of the model species within such habitats, also in managed forests. There is already evidence that the effective conservation-oriented management of this species should be tailored at the habitat level, and especially at the tree level, where microclimatic features are of utmost importance [5,15]. Therefore, it is essential to understand microscale dynamics under various microclimatic conditions to be able to design effective conservation strategies suitable to all stages of population development [15].

In a previous study [20], comparing young and adult *L. pulmonaria* thalli exposed for one year in logged and unlogged stands (on the northern side of Turkey oaks), a lower growth of *L. pulmonaria* was observed on isolated trees in the logged stand, especially in the case of adult thalli. Similarly, native thalli on isolated trees appeared thinner and showed lower photosynthetic performance and water holding capacity when compared with healthy samples from unlogged forests or retained forest patches [21], with consequent implications for their growth and the possibility of providing ecosystem services.

The translocation of lichen thalli (or fragments of thalli) can be regarded as a method for the in situ conservation of threatened lichen populations [22]. Some projects have investigated this opportunity, monitoring the performance of translocated *L. pulmonaria* thalli [23]. The aim of the present work was to investigate the combined effect of forest logging and microclimatic conditions on the probability of survival of transplants of the model species. Our working hypothesis is that microclimatic alteration at the tree level after logging affects their growth, with young thalli having a higher chance of survival. 

To this purpose, 800 fragments of *L. pulmonaria* were transplanted in logged and unlogged stands for two years. Fragments were positioned on oak trunks according to the distance from the ground (100 and 50 cm) and aspect (north and south). Since the viability of *L. pulmonaria* populations in relation to forest management often depends on the regenerative capacity of young and adult thalli, two types of fragments were considered: those with meristematic (young) properties, and those with non-meristematic (adult) properties. Growth rates and probabilities were assessed and modelled for each experimental condition.

## 2. Results

### 2.1. Fragment Growth

After two years of exposure, transplanted fragments presented a wide range of growth rates, with an average growth rate of 16%. In detail, exposed fragments in the unlogged stand showed a higher growth rate (31%) when compared to exposed fragments on isolated trees in the logged stand (10%). North-exposed fragments grew more (26.9%) than those exposed to the south (14.7%). Similarly, meristematic lobes presented higher percentages of growth (25.8%) than adult fragments (16%). By contrast, only a slight difference emerged between fragments exposed at 100 cm (22%) and 50 cm (19%) from the ground. Regarding the growth of the fragments, only 44% had positive growth. South-exposed fragments in the logged stand had the lowest rate of positive growth (4%). Most of these fragments appeared progressively damaged during the transplant period, and were finally lost, having fallen or broken apart.

### 2.2. Determinants of Probability and Coefficient of Growth

**Figure 1:**
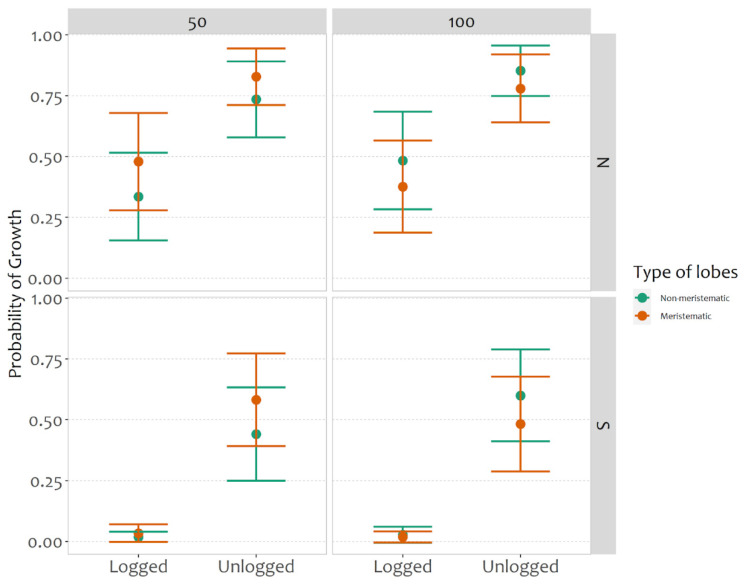
Predicted probability of growth for forest types (logged and unlogged), type of lobes (non-meristematic and meristematic), aspect (N = north, S = south) and different heights from the ground (50 and 100 cm).

Models considering the probability of growth of the fragments highlighted a lower growth probability in the logged stand and southern aspect (Figure 1, Table 1). They also revealed a significant interaction between the forest stands and their aspect (Figure 1, Table 1), with fragments exposed to the south in the logged stand showing low probability of growth. In addition, the interaction between type of lobes and height from the ground was also significant. Higher growth probabilities were associated with the unlogged stand, particularly for north-exposed transplants. Meristematic fragments exposed at 50 cm from the ground had a higher chance of growth; by contrast, non-meristematic fragments showed higher growth probability at 100 cm.

**Figure 2:**
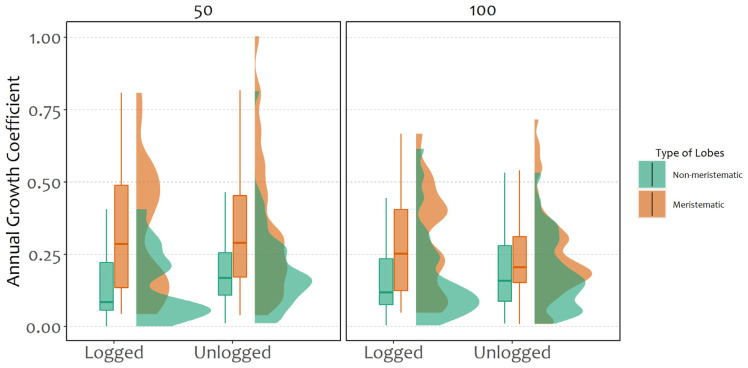
Annual growth coefficient for forest type (logged and unlogged), type of lobes (non-meristematic and meristematic) and different heights from the ground (50 and 100 cm).

Modelling related to growth coefficients (Figure 2, Table 1) indicated a significant difference between meristematic and non-meristematic fragments, with the first having a higher growth coefficient. Considering the whole dataset, no significant correlation between forest type and distance from the ground was evident, suggesting an overall similar growth both in logged and unlogged stands, regardless of the distance from the ground (Figure 2, Table 1). However, it is important to state that such models considered only fragments that had a positive growth coefficient, not including aspect as a predictive variable, since few fragments presented a positive growth coefficient in the southern side of the trees in the logged stand.

**Figure 3:**
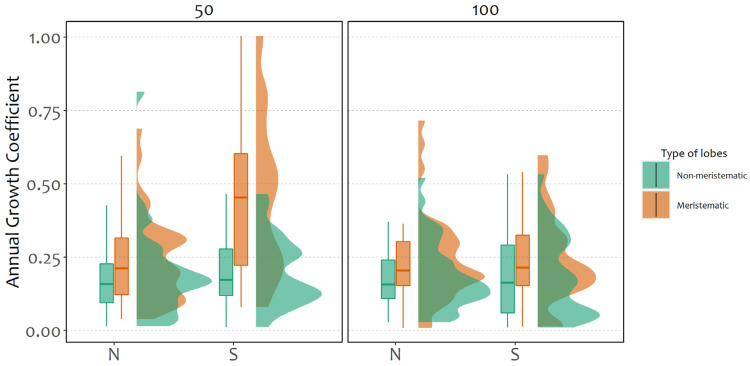
Annual growth coefficient in the unlogged stand according to type of lobes (meristematic and non-meristematic), aspect and different heights from the ground (50 and 100 cm).

Therefore, focusing specifically on growth coefficients in the unlogged stand, models outlined higher growth by meristematic fragments (Figure 3, Table 1) and moreover, south-exposed fragments had a higher growth coefficient than those that were north-exposed, especially at 50 cm from the ground.

## 3. Discussion

Our results support the hypothesis that microclimatic conditions are extremely important in determining the growth of *L. pulmonaria* transplants, and highlight the relevance of considering microclimatic factors when performing forest logging in areas hosting populations of *L. pulmonaria*. Preserving retained forest patches as well as isolated trees growing in favorable microclimatic conditions can maximize the possibility of *L. pulmonaria* populations persisting, even after forest logging [21]. In fact, microclimatic refugia can reduce species threat if suitable climatic conditions are maintained locally, regardless of processes at the larger scale that modify environmental conditions [1].

Due to their small living space, lichens are strongly influenced by local microclimatic variations. In general, the physiological responses, abundance and diversity of smaller and less mobile organisms often more readily reflect small-scale variations in micro-environmental conditions than do the responses of organisms characterized by larger living space, size and mobility [2,24]. After forest logging, microclimatic conditions drastically change, with a strong increase of temperature, light and wind speed, and a reduction of humidity [25]. Understanding the effects of the main microclimatic [this study, 18] and macroclimatic [26] drivers on the survival and growth of *L. pulmonaria* is necessary in order to boost the capability of this species to recolonize fragmented or disturbed habitats and to develop proper conservation strategies. This also holds true in the case of translocations for conservation purposes, the success of which requires specific protocols [27] and depends on the selection of receptor sites with locally suitable microclimatic and chemical conditions that allow the long-term persistence of translocated thalli and the development of new ones [22,28].

In our study, fragments exposed to the south in the logged stand had an extremely low probability of growth, being progressively damaged during the transplant period and finally falling or breaking after two years. In the northern hemisphere, without considering other factors, south-exposed sites tend to receive higher amounts of light and are drier compared to north-exposed sites. This phenomenon can be exacerbated by forest logging, which, by reducing canopy cover, causes an increase in light intensity and reduces the thermal buffer capacity of the forest [25].

*Lobaria pulmonaria* is susceptible to high light stress, particularly in a desiccated state [16,29,30]. Moreover, the combined effect of high temperature and irradiance can be particularly deleterious to the thalli [16]. In our study, the microclimatic conditions of the fragments exposed to the south in the logged stand likely exceeded the ecological range of conditions suitable for acclimation. The combination of high temperature and irradiance, as well as limited water availability, especially characterizes open south-facing sites during the summer in Mediterranean areas [31]. Solar radiation strongly influences fragment surface temperature, enhancing water loss and reducing the total hydration period [32]. Hence, south-exposed fragments could only find limited favorable periods for photosynthesis and growth, leading to the survival of almost no fragments after two years. Nevertheless, like many other organisms, *L. pulmonaria* possesses different mechanisms for acclimation, which, within a certain range, allows it to withstand changes in microclimatic conditions after forest logging. As an example, the species is able to adapt to high light stress through the melanisation of the thallus [33], as already observed as a response to logging, or in transplant experiments [20,21,34]. *Lobaria pulmonaria* synthesizes melanins when exposed to high solar radiation; this melanisation requires sufficient hydration of the thallus to occur and may effectively reduce high light stress by increasing energy dissipation and reducing photosynthetic activity [33]. Furthermore, by increasing thallus thickness the lichen can prolong hydration periods and can also prolong photobiont protection [35]. Acclimation strategies can be observed at different levels and involve gene expression, for example, to acclimate to thermal shifts [36], or during water dehydration cycles [37]. 

In our study, the coefficient of growth of those fragments still attached after two years highlighted only minor differences between the logged and the unlogged stands. These results suggest that in the logged stand, certain microclimatic conditions can resemble those present in the retained forest, allowing fragment growth. Specifically, north-exposed fragments in the logged stand received far lower amounts of light than south-exposed fragments, with the first benefitting from the shading effect of the tree. Forest gaps and clear-cuts are characterized by frequent dew events [32,38], which often occur during the night, especially at the northern side, so north-exposed thalli can benefit from them, prolonging their hydration periods. Hence, the trees’ shading effect combined with these dew events could explain the lack of difference in terms of growth coefficients between north-exposed fragments in the logged stand and fragments in the unlogged stand. A hydrated state of the thallus is essential for surface growth in lichens, as turgor pressure is required for hyphal expansion [39].

Previous studies in boreal forests have reported better growth of lichens in sheltered clear-cuts than in intact old forests [35,40]. However, in boreal forests, clear-cuts receive less solar radiation overall and reach lower temperatures compared with Mediterranean oak forests, where high irradiance is accompanied by a rise in temperature and an overall decrease of moisture. Consequently, in our study sites, north-exposed fragments on isolated trees (despite finding favorable conditions to grow compared to south-exposed fragments), were not in better conditions than those in mixed unlogged stands. 

Considering the results from the unlogged stand, aspect seemed to play a relevant role in combination with distance from the ground. South-exposed fragments could have received slightly more light than those exposed to the north. Unlike isolated trees, this increase in the amount of light could have enhanced lichen growth.

The distance from the ground seemed to influence growth coefficients only in the unlogged stand, while showing negligible effects in the logged stand. This result is consistent with a previous study [41], which reported that in open Mediterranean forests, the fast growth of *L. pulmonaria* near the ground is likely boosted by nocturnal cooling forming temperature profiles, and hence, humidity gradients. Thus, the effect of height on lichen growth rates depends highly on climate and forest type [41] and in our case, a fine combination of increased southerly light together with sufficient humidity in the unlogged stand led to an overall higher growth coefficient, especially for south-exposed fragments at 50 cm from the ground. 

The type of lobes was a relevant factor in determining *L. pulmonaria* growth rates. Meristematic fragments grew on average more than non-meristematic ones, regardless of aspect. Similarly, our previous study highlighted that north-exposed meristematic fragments measured after one year had higher growth rates (0.16–0.18 cm^2^) than non-meristematic ones (0.02–0.06 cm^2^), irrespective of forest management (i.e., comparing logged versus unlogged stands) [20]. On the other hand, the results also point out the capacity of both meristematic and non-meristematic tissues to regenerate and grow, even after severe damage, as previously observed for other lichen species [42].

In this sense, small fragments of *L. pulmonaria* that remain after logging could be a potential source of new propagules which, when favorable conditions occur, could allow the development of new populations, with faster growth of young (meristematic) thalli. Hence, in Mediterranean oak forests, retaining trees where *L. pulmonaria* colonize the northern side can greatly enhance the possibility of those thalli persisting even after logging. Nevertheless, taking a long-term view, the spatial distribution of retained trees also has to be considered, as the vegetative dispersal capacity of *L. pulmonaria* has a low distance [43,44]. 

As concluding remarks, since the transplant technique, in the case of *L. pulmonaria*, can be used for conservation purposes via translocation of the thalli [22,28], we should consider the following in relation to Mediterranean oak forests:

- The translocation on isolated trees (e.g., in unlogged stands) better supports the survival of thalli on the northern side of the boles, given the stronger sun irradiance and lower water availability at the southern side.
- The translocation on trees within forest patches or unlogged stands offers higher growth performance on the southern side of the trees, thanks to the fine combination of increased light on the southern side and sufficient humidity within the forest.

Constraints such as microclimate are fundamental considerations when dealing with the ecology of lichens. They open up the opportunity to protect valuable habitats and local populations, and in general, to adapt forest management practices for the conservation of threatened species.

## 4. Materials and Methods

### 4.1. Study Area and Experimental Design

The study area was a Mediterranean mixed oak forest stand dominated by *Quercus cerris*, *Q. pubescens* and *Q. ilex* recognized as a local hotspot for *L. pulmonaria* [45] (Tuscany, Central Italy, WGS84: N 43.1851; E 11.3602). Oak forests in the area are managed by a coppice system with a long rotation cycle. The last cut was carried out in 2016 (the previous cut likely dates back more than 40 years). Logging reduced the density of stems from ~1100 to 165 per ha, with a consequent increase of sun irradiance (from 130–1100 in the unlogged stand to 900–1550 μmol m^−2^ s^−1^ PAR, measured at noon in the logged stand) and temperature, as well as a decrease of humidity [21]. Since *L. pulmonaria* is not legally protected in Italy, logging operations started without considering the presence of this relevant population, which resulted heavily impacted by logging [7].

**Figure 4:**
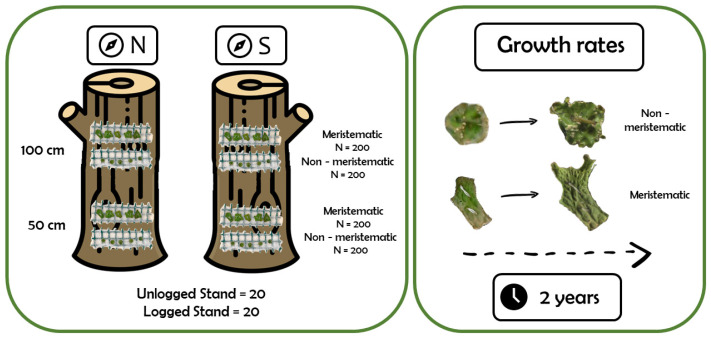
Graphical representation of the experimental design. Icons: Flaticon.com.

According to the methodology described in [20], healthy *L. pulmonaria* thalli were randomly selected from a nearby oak forest to obtain 400 meristematic fragments and 400 non-meristematic fragments (Figure 4). Specifically, the first were upward-growing young lobes with intact apical meristems, and the latter were fragments of the inner sorediate or non-sorediate parts of the thallus, lacking apical growth [46]. The source habitat for collected fragments had the same characteristics as the unlogged stand, being adjacent to the study sites and extending onto a hillside with a northern slope, where *Q. cerris*, *Q. ilex* and *Q. pubescens* were the most common trees colonized by *L. pulmonaria* [20]. In order to minimize the harvesting of material from the native population, all fragments were smaller than 1 cm. Overall, 800 fragments of *L. pulmonaria* were transplanted for two years (from March 2019 to March 2021). Thallus fragments (each representing a statistical sample) were exposed on the northern and the southern sides of the trunks of twenty randomly selected Turkey oaks (*Q. cerris*) (reciprocal distance >10 m), at about 100 cm and 50 cm from the ground, half in the logged and half in the unlogged stand: 400 meristematic and 400 non-meristematic fragments in each forest type (logged and unlogged stands). *Lobaria pulmonaria* was exposed using a specific transplant device, a ‘barella’, which was composed of a sterilized bandage supported by a plastic net (10 × 2 cm^2^). For each device, for practical reasons, five meristematic or five non-meristematic fragments of thalli were tied onto the bandage to avoid overlapping with each other [20].

### 4.2. Growth

The hydrated thallus area (A) was estimated using Photoshop CS6 Extended (Adobe Systems, San Jose, CA, USA), as suggested by [20]. To avoid folding of the lobes, each thallus fragment was fully hydrated with mineral water and carefully flattened before scanning with a Canon i-SENSYS MF4320d (Canon Inc., Tokyo, Japan). The lichen growth, comparing the same samples before (T_0_) and after (T_F_) exposure, was estimated as growth coefficient A = (area T_F_ − area T_0_)/area T_0_ and as percentage of growth A = [(area T_F_ − area T_0_)/area T_0_] × 100. After two years, the growth of the surface of each individual thallus was assessed by subtracting the initial area from the respective area at harvest. Pre-exposure and final values were considered for the growth rates.

### 4.3. Data Analysis

Each fragment was categorized as follows: (a) fragments with a positive coefficient of growth (growth coefficient >0) were defined as ‘growth’; and (b) fragments with a negative coefficient of growth (<0), together with those that were completely degraded (growth coefficient = −1), were defined as ‘no growth’. Then, the effects of forest type (logged versus unlogged), aspect (N versus S), height from the ground (50 versus 100 cm) and type of lobes (meristematic versus non-meristematic) were tested together with their possible interactions on the probability of growth. A generalized linear mixed model (GLMM) was fitted with ‘tree’ as a random factor to account for possible similar unmeasured conditions on the same tree and a binomial distribution with logit link function.

Subsequently, only those fragments with a positive growth coefficient were selected, and the effect of forest type, height from the ground, type of lobes and their interactions on growth coefficient were modelled. We did not include aspect as a predictive variable, since in the logged stand only nine fragments exposed to the south presented positive growth, thus making unsuitable for testing. We fitted a linear mixed model (LMM) with ‘tree’ as a random factor. Growth coefficients were treated with the square root to meet normality.

Finally, a model was fitted using only those fragments with positive growth coefficients in the unlogged stand. In such cases, the effects of exposure, height from the ground, type of lobes and their interactions on growth coefficient were tested. An LMM was fitted with ‘tree’ as a random factor, an in this case, growth coefficients were also treated with the square root.

All models were fitted using the ‘glmmTMB’ package [47]. The performance of the models was checked through diagnostic plots and tests using the ‘DHARMa’ package [48].

Predictor importance was evaluated using multimodel inference through the information-theoretic approach [49]. All candidate models, nested within the full model, were fitted and compared using Akaike’s information criterion corrected for sample size (AICc). Models were ranked based on their difference in AICc (ΔAICc) with respect to the best-fitting model (i.e., the one with the lowest value of AICc, AICc min). Thus, ΔAICc was calculated as ΔAICc*i*  =  AICc*i*  −  AICc min. Models that presented a ΔAICc < 2 were considered plausible, and the coefficient of each predictor was averaged among this set of models. Then, the significance of the estimated coefficients was tested using a z-Wald test. For each predictor, its relative importance ‘*wr*’ was calculated by summing the AICc weights of all the models where that predictor appeared [50]. AICc weight, the relative likelihood of a specific model, was calculated by dividing the likelihood of the model by the sum of likelihoods of all models. The functions *dredge*, *model.avg* and *importance* were used in the MuMIn package to perform the multimodel inference [51]. Graphical outputs were produced using ggplot2 [52] and ggdist [53] packages. All analyses were performed using R 4.1.1 [54].

## Figures and Tables

**Table 1:** Full averaging of generalized linear mixed model coefficients among models showing a delta AICc < 2. Significant interactions are in bold. Variables with sum >0.65 and with a significant Wald test are shown in bold.

Parameter	Relative Importance	Lower CI	Upper CI	Full Averaged Coefficient	*p*-Value
**Probability of growth**					
Height from the ground	1.000	−0.246	0.173	−0.037	0.731
Aspect	**1.000**	**0.927**	**1.413**	**1.170**	**0.000**
Type of lobes	1.000	−0.214	0.150	−0.032	0.732
Forest type	**1.000**	**−1.898**	**−0.884**	**−1.391**	**0.000**
Height from the ground: Type of lobes	**1.000**	**−0.442**	**−0.079**	**−0.261**	**0.005**
Aspect: Forest type	**1.000**	**0.282**	**0.763**	**0.523**	**0.000**
Height from the ground: Forest type	0.346	−0.272	0.101	−0.013	0.791
Height from the ground: Aspect	0.330	−0.102	0.373	0.047	0.625
Type of lobes:Forest type	0.147	−0.366	0.114	−0.041	0.651
Aspect:Type of lobes	0.107	−0.142	0.221	0.004	0.897
Height from the ground: Aspect: Forest type	0.103	−0.425	0.072	−0.018	0.788
**Coefficient of growth**					
Height from the ground	1.000	−0.009	0.033	0.012	0.269
Height from the ground: Type of lobes	0.637	−0.032	0.003	−0.009	0.356
Height from the ground: Forest type	0.425	−0.038	0.004	−0.007	0.505
Type of lobes	**1.000**	**−0.083**	**−0.040**	**−0.062**	**0.000**
Type of lobes:Forest type	0.581	−0.039	0.001	−0.011	0.366
Forest type	0.752	−0.053	0.015	−0.014	0.403
**Coefficient of growth—Only unlogged**					
Height from the ground	**1.000**	**0.010**	**0.048**	**0.029**	**0.003**
Aspect	**1.000**	**−0.051**	**−0.011**	**−0.031**	**0.002**
Type of lobes	**1.000**	**−0.068**	**−0.031**	**−0.049**	**0.000**
Height from the ground: Aspect	**1.000**	**−0.048**	**−0.010**	**−0.029**	**0.003**
Aspect:Type of lobes	1.000	−0.035	0.004	−0.010	0.343
Height from the ground: Type of lobes	**0.671**	**0.003**	**0.041**	**0.022**	**0.025**
Height from the ground: Aspect: Type of lobes	0.371	−0.003	0.035	0.006	0.546
